# A propos de 39 cas de diverticules sous-urétraux de la femme: expérience monocentrique sur 30 ans

**DOI:** 10.11604/pamj.2023.46.51.41246

**Published:** 2023-10-06

**Authors:** Salim Lachkar, Ahmed Ibrahimi, Imad Boualaoui, Hachem El Sayegh, Yassine Nouini

**Affiliations:** 1 Service d’Urologie A, Centre Hospitalier Universitaire Ibn Sina, Rabat, Maroc

**Keywords:** Diverticule sous-urétral, infections urinaires récidivantes, diverticulectomie transvaginale, Suburethral diverticulum, recurrent urinary tract infections, transvaginal diverticulectomy

## Abstract

Nous rapportons l´analyse rétrospective de 30 années d´expérience concernant 39 femmes atteintes de diverticule sous-urétral. L´âge moyen est de 37 ans (24-56 ans). La parité moyenne est de 2 (1-7). Soixante-cinq pourcent (65%) soient 25 des accouchements sont dystociques, avec utilisation de forceps dans 43% (17) des cas. Des antécédents infectieux urologiques ou gynécologiques sont présents chez toutes les patientes. Les symptômes révélateurs sont hétérogènes et sont principalement les infections urinaires récidivantes (26 cas), la pollakiurie (23 cas), l´écoulement urétral post-mictionnel (21 cas), la douleur vaginale (17 cas) et une sensation de boule vaginale (15 cas). Le bilan radiologique est variable: urographie intra-veineuse, urétrocystographie rétrograde et mictionnelle, échographie ou IRM. La diverticulectomie par voie transvaginale est le traitement pour toutes les patientes, sans complication per-opératoire rapportée. A 4 ans les résultats sont satisfaisants. Nous déplorons 4 récidives diverticulaires. Ces données fournissent des informations importantes sur les caractéristiques cliniques, les résultats diagnostiques et les résultats à long terme de la diverticulectomie transvaginale, permettant ainsi une meilleure prise en charge de cette affection rare.

## Abstract (en)

We here report a retrospective analysis of 30 years´ experience with 39 female patients suffering from suburethral diverticula. The average age of patients was 37 years of age (24-56 years). The average parity was 2 (1-7); 65% of deliveries were complicated by dystocia, with forceps used in 43% of cases. All patients had a history of urological or gynaecological infections. The revealing symptoms were heterogeneous but recurrent urinary tract infections (26 cases), pollakiuria (23 cases), post-micturition urethral discharge (21 cases), vaginal pain (17 cases) and a sensation of vaginal bulge (15 cases) were mostly reported. Radiological assessment were performed, including intravenous urography, retrograde and micturition urethrography, ultrasound, or MRI. Transvaginal diverticulectomy was the treatment of choice for all patients, with no reported intraoperative complications. At 4 years of follow up outcome was satisfactory. Four patients developed recurrence of diverticulitis. These data provide important information about clinical features, diagnostic results and long-term outcomes of transvaginal diverticulectomy, enabling better management of this rare condition.

## Introduction

Le diverticule sous urétral est une pathologie rare. Son incidence dans la population féminine est estimée entre 0,6% et 6%. Il est exceptionnellement rapporté chez l´homme et l´enfant [1]. Il s´agit d´une hernie de la muqueuse urétrale à travers les fibres musculaires lisses. Le sac herniaire communique avec la lumière urétrale par un collet et fait protrusion au niveau du septum inter urétro-vaginal [2]. Son etiopathogénie est mal connue. Il peut être congénital ou acquis [3]. A côté des formes asymptomatiques, la survenue de symptômes du bas appareil urinaire, surtout obstructif, est le principal mode de révélation [4]. Le diagnostic est clinique [2]. La cure chirurgicale du diverticule ou diverticulectomie est le traitement de référence [5]. Le cadre scientifique de notre étude vise à comprendre l´incidence et les caractéristiques du diverticule sous urétral chez les femmes de notre population locale. Nous souhaitons identifier les facteurs de risque associés à sa survenue, évaluer les manifestations cliniques et étudier les options de prise en charge appropriées. Nos hypothèses préalables sont que l´incidence du diverticule sous urétral dans notre population féminine locale est similaire aux estimations internationales, certains facteurs comme l´âge ou les antécédents obstétricaux peuvent accroître le risque, les symptômes obstructifs du bas appareil urinaire seront plus fréquents chez les femmes atteintes de diverticules sous urétraux et la diverticulectomie améliorera les symptômes urinaires et réduira les récidives.

## Méthodes

**Cadre de l´étude:** il s´agit d´une étude rétrospective descriptive des dossiers médicaux de 39 patientes prises en charge dans notre service pour un diverticule de l´urètre entre janvier 1986 à décembre 2022. Il s´agit d´une étude exhaustive regroupant tous les cas pris en charge dans notre formation.

**Type d´étude:** il s´agit d´une série de cas.

**Participants à l´étude:** nous avons recruté nos patientes selon les critères d´inclusion suivants: patiente de sexe féminin, porteuse d´un diverticule sous urétral confirmé et prise en charge dans le service d´Urologie A du CHU Ibn Sina de Rabat durant la période 1986-2022.

**Conception de l´étude:** nous avons collecté et exploité les différentes données épidémiologiques, cliniques, anatomopathologiques et thérapeutiques de nos patientes à partir de leurs dossiers médicaux, puis nous avons comparé ces données à la littérature en utilisant les bases de recherches de PubMed, Scopus et Google Scholar.

**Analyse des données:** les données présentées dans le texte sont principalement observationnelles avec des informations descriptives. Il n´a pas été utilisé de méthodes statistiques formelles.

**Consentement éclairé:** nous avons obtenu le consentement éclairé de toutes les participantes que nous avons pu contacter, conformément aux normes éthiques et réglementaires. Le processus de consentement a été expliqué en détail, garantissant ainsi la participation volontaire et informée de chaque individu. La nature rétrospective de nos données a parfois rendu difficile la joignabilité de certains patients.

## Résultats

**Caractéristique des patientes:** l´âge moyen est de 37 ans (24-56 ans) quatre patientes sont nullipares. La parité moyenne est de 2(1-7). 65% des accouchements ont été au moins une fois dystocique et 43% ont nécessité l´utilisation de forceps. Dans tous les cas des antécédents infectieux urologique ou gynécologiques sont retrouvés. Dans 87% des cas il s´agit de cervicovaginite ou de salpingite. Le délai moyen de consultation est de 20 mois (2 mois-7 ans).

**Figure 1:**
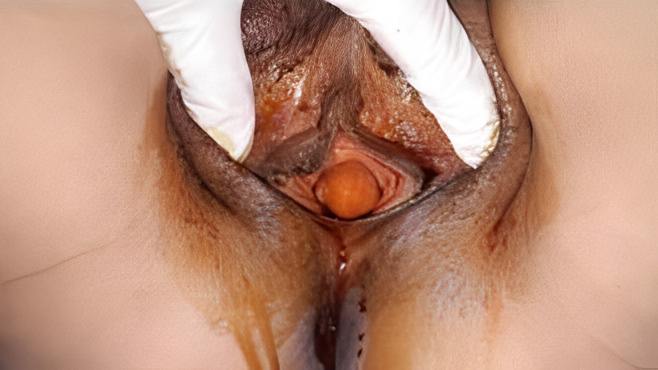
vue pré-opératoire d´un diverticule sous urétral

**La symptomatologie révélatrice est hétérogène:** infections urinaires récidivantes (26 cas), pollakiurie (23 cas), écoulement urétral post mictionnel (21 cas), douleur vaginal (17 cas), perception d´une voussure vaginale (15 cas), dysurie (10%), dyspareunies (2 cas), rétention aigue des urines (2 cas) et hématurie intermitante terminale (2 cas). Dans tous les cas l´examen clinique met en évidence une masse vaginale antérieure de consistance molle, avec émission d´urines purulentes à la pression (20 cas) Figure 1. Le diverticule est majoritairement unique (34 cas).

**Figure 2:**
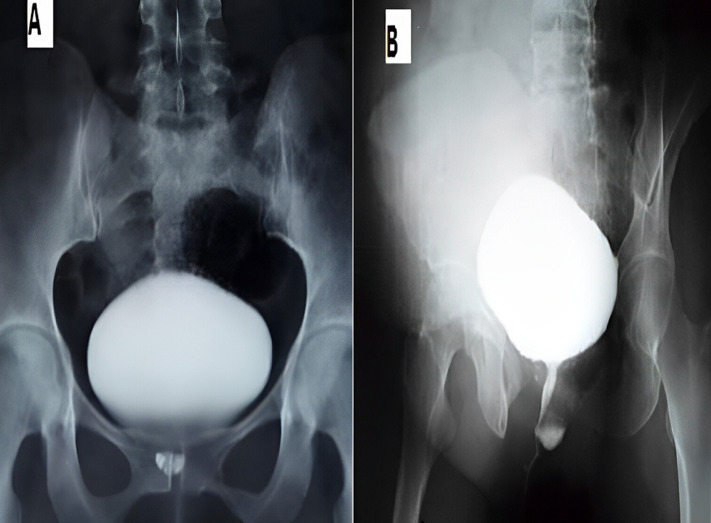
résultat d´une uretrocytographie rétrograde pour diverticule

**Figure 3:**
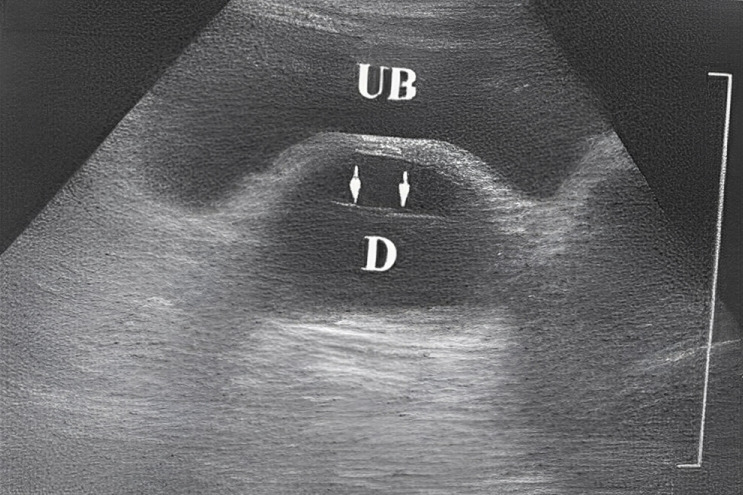
résultat d´une échographie pour diverticule UB-vessie, D-diverticule

**Figure 4:**
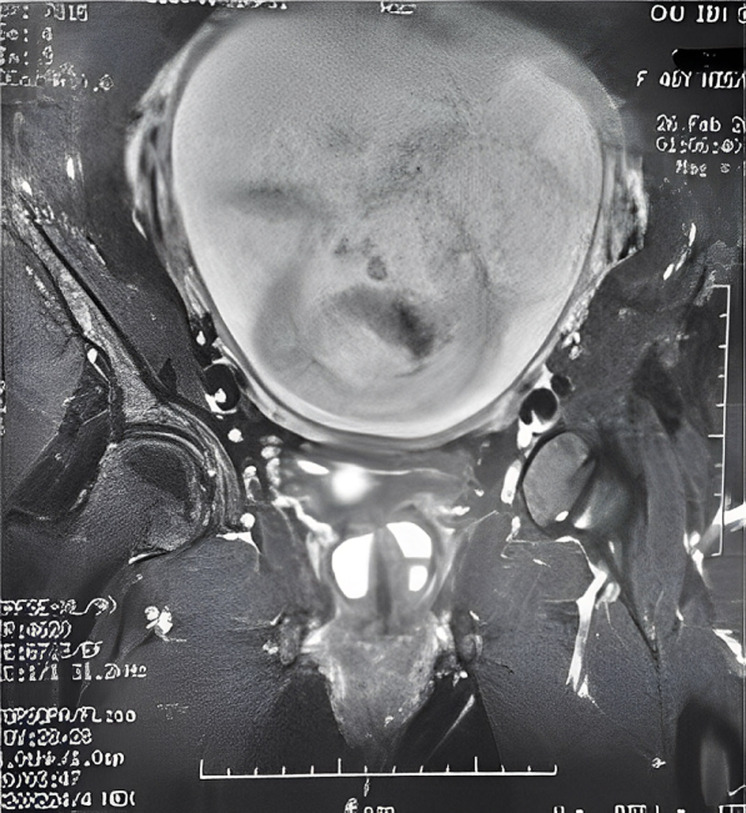
résultat d´une IRM pour diverticule

**L´imagerie:** l´urographie intra-veineuse UIV (8 cas), montre dans 5 cas une opacité arrondie de la région sous-vésicale sur le cliché post-mictionel. Une urétrocystographie rétrograde et mictionelle UCRM (26 cas), montre à chaque fois une opacité arrondi siégeant derrière l´urètre (Figure 2). Une échographie transpérinéale (15 cas), montre à chaque fois une image arrondie hypoechogène, parfois hétérogène avec un sédiment urinaire (Figure 3). L ´imagerie par résonnance magnétique pelvienne IRM est faite dans un cas douteux (Figure 4). L´urétro-cystoscopie (18 cas) retrouve dans 15 cas le collet diverticulaire. L´examen cytobactériologique des urines est systématique. Il est positif dans 34 cas: *Escherichia Coli* (16 cas), *Klebsiella*.

**Figure 5:**
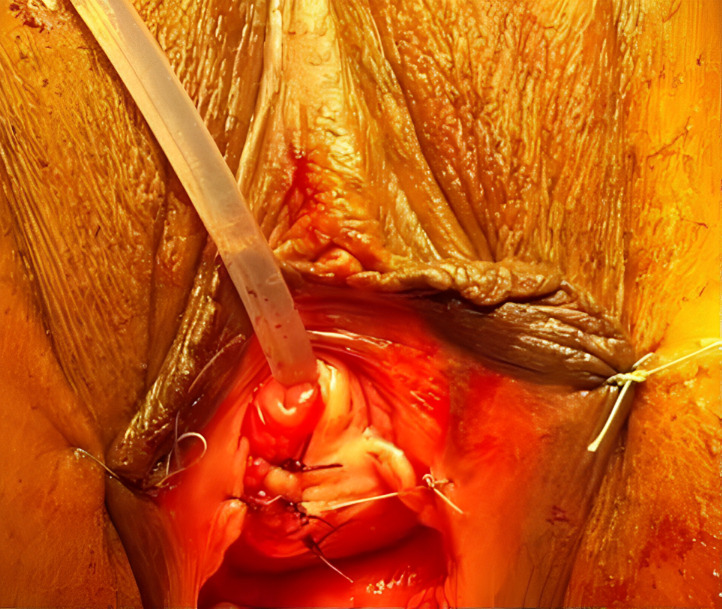
vue post-opératoire immédiate

**Le traitement est chirurgical dans tous les cas:** diverticulectomie par voie trans-vaginale, sous rachianesthesie, en position genu-pectorale, avec incision vaginale antérieure longitudinale (27 cas) ou en U inversé (12 cas) (Figure 5).

**Complication et suivie:** aucune complication per-opératoire n’est rapportée, l´ablation de la sonde urétrale est faite vers le 4^e^ jour en moyenne. L´étude anatomopathologique du sac herniaire est systématique et à chaque fois sans particularité. Le suivi médian est de 4 ans, avec des résultats satisfaisant et disparitions des signes cliniques (35 cas). Dans 4 cas il y a une récidive du diverticule.

## Discussion

Le diverticule sous-urétral de la femme est rare, son incidence varie de 0,6 à 6% [1]. Il survient souvent entre 30 et 50 ans [2], en concordance avec les données de notre série. L´ethiopathogénie est mal connue, avec une origine probablement multifactorielle. Une incidence accrue allant jusqu´à 6 % dans les populations afro-américaines suggère l´implication de facteurs génétiques héréditaires [3]. L´existence de diverticules sous urétraux chez le nouveau-né sous-tend une origine congénitale. Le diverticule est alors soit isolé soit associé à un syndrome polymalformatif de l´appareil urogénital: reliquats mésonéphrotiques (canal de Gardner), anomalies d´accolement des bourgeons embryonnaires primitifs (union défectueuse des plis primaires), kystes de la paroi vaginale d´origine müllérienne ou dilatation congénitale des glandes para-urétrales [2]. L´origine acquise reste la plus fréquente [4]. Plusieurs facteurs de risque sont décrits: multiparité, traumatisme obstétrical (forceps) ou sténoses urétrales [3]. Il existe une corrélation étroite entre infections urogénitales et diverticules sous urétraux, le diverticule étant parfois un abcès des glandes peri-urétrales fistulisées dans l´urètre [2]. Plusieurs de ces facteurs sont largement retrouvés dans notre série. La symptomatologie est polymorphe. Il s´agit le plus souvent de la tétrade des “4D”: douleur urétrale violente (souvent en fin de miction), dribbling, dyspareunie et dysurie [4], tous retrouvés dans notre série. L´issue de pus ou urine par l´urètre lors de rapports sexuels et les infections urinaires récidivantes sont également très évocateurs [3]. L´association à incontinence urinaire d´effort est inconstamment retrouvée de 5 à 71% des cas [5].

L´urétrorragie et la rétention aiguë d´urine sont plus rarement retrouvées [4]. Dix (10) à 20% des diverticules restent asymptomatiques. L´examen clinique sous valve pose le diagnostic [2], révélant une tuméfaction molle des deux tiers distaux de l´urètre, postéro-latéral, rarement en antérieure [3]. La présentation est hétérogène: diamètre variant de quelques millimètres à plusieurs centimètre, unique ou multiple, collet diverticulaire punctiforme ou centimètrique [1]. L´écoulement de liquide par le méat urétral à la pression douce est caractéristique, rapporté par 45% de nos patientes, en concordance avec la littérature [4]. Une induration diverticulaire oriente vers des calculs ou une tumeur intradiverticulaire [4]. L´urétrocystographie rétrograde et mictionelle est un examen de référence. Sa sensibilité est de 85% [6]. Il montre typiquement une opacité arrondie retro-urétrale et objective l´éventuel caractère rétentioniste du diverticule [6]. Dans les cas douteux, l´urétrographie à pression positive avec sonde urétral à double ballon double la sensibilité de l´examen [7]. La diverticulographie par ponction directe est décrite [3]. L´échographie est plus sensible mais moins spécifique. Elle étudie au mieux les diverticules non opacifiés. Plusieurs techniques sont décrites: sus-pubienne, trans-périnéale et trans-labiale, endo-vaginale ou endo-uretrale. L´echographie dynamique du plancher pelvien supplante actuellement l´uretrocystographie [8]. L´IRM pelvienne avec injection de Gadolinium a une sensibilité proche de 100%, même en cas de petit diverticules. Elle est réservée aux cas douteux [6]. L´utilisation de la cystographie rétrograde et mictionelle avec acquisition par tomodensitométrie ou 16-MDCT *(Multiple Detector Computed Tomography cystoscopy)* est rapporté [9].

L´urètro-cystoscopie recherche le collet diverticulaire et une tumeur intra-diverticulaire. Jusqu´à 30% des cystoscopies échouent à retrouver l´orifice diverticulaire [5]. Les diagnostics différentiels sont résumés dans le Tableau 1 [1]. Les complications infectieuses du diverticule sont très fréquentes, jusqu´à 60%, allant des infections urinaires à répétitions aux suppurations intra-diverticulaires et fistulisation intra-vaginale [2]. La présence de calculs intra-diverticulaires est variable, de 1,5 à 34% des cas [1]. Un cas est retrouvé dans notre série. Moins de 100 cas de tumeur maligne intradiverticulaire sont rapportés: adénocarcinome (60%), carcinome urothéliaux (28%) et carcinome épidermoïde (12%). Quinze (15) cas d´adénome néphrogénique bénin sont retrouvés [10]. Le traitement de référence est chirurgicale: la diverticulectomie transvaginale [4]. L´application préalable d´œstrogène locaux semble améliorer la trophicité des tissus. La position genu-pectorale est la plus fréquente. Le décubitus ventral est possible [2]. La distension préalable du diverticule facilite la dissection: sonde urétérale enroulée dans le diverticule, sonde de Fogarty ou injection de sillicone [4]. La préservation du fascia péri-urétral améliore les résultats à long terme. L´interposition d´un lambeau graisseux des grandes lèvres entre le facia péri-urétrale et la paroi vaginale est possible en cas de tissus péri-urétraux de mauvaise qualité [11]. L´utilisation de la laparoscopie robot assistée est décrite, sans supériorité vis-à-vis de la voie transvaginale classique [12]. En cas d´impossibilité de la diverticulectomie, des techniques alternatives existent. Elles améliorent uniquement le drainage du diverticule. Il s´agira de faire communiquer le diverticule et le vagin *(marsupialisation vaginal de Spence et Duckett)* [13] ou d´inciser endoscopiquement le collet diverticulaire (*technique de Lapides*) [14].

**Tableau 1:** diagnostiques différentiels des diverticules sous urétraux de la femme [1]

Abcès des glandes de Skène	Néoplasie urètrale	Hémangiome
Kyste du canal de Gardner	Néoplasie vaginale antérieure	Varices urétrales
Kyste mullérien		
Urétérocèle ectopique	Fibromyome péri urétral	Endométriose urétrale
Urétrocèle	Fibromyome vaginal	
Cystocèle vaginal		
Kyste de la paroi vaginale		

D´autres méthodes sont décrites: éléctrocoagulation trans-urétrale, comblement par compresses de cellulose , excision partielle du diverticule ou injection intradiverticulaire de Téflon [4]. L´incidence des complications de la diverticulectomie varie de 8 à 20%. Elles sont résumées dans le Tableau 2 [1].

**Tableau 2:** complications des diverticules sous urétraux de la femme [1]

Complication	Pourcentage (%)
Fistules urétro-vaginales	6
Récidive du diverticule	15
Incontinence urinaire	11
Fistules cervico-vaginales	rare
Sténoses urétréales	rare
Infection urinaire	2

La diverticulectomie donne le meilleur taux de succès: 89 à 96% contre 78% pour la marsupialisation vaginale [15]. Nous avons réalisé une diverticulectomie pour toutes nos patientes.

**Limites:** l´étude présente certaines limites: a) la population de 39 patientes limite la généralisation des résultats; b) la nature rétrospective induit de possible biais de sélection et des limitations dans la collecte et l´analyse des données; c) les données utilisées ont été extraites des dossiers médicaux, avec de possible lacunes dans les informations disponibles.

## Conclusion

Le diverticule sous-urétral de la femme est une affection rare chez les femmes d´âge moyen. Il est à l´origine de nombreuses manifestations urogénitales polymorphes. Il doit être systématiquement recherché en cas d´infections urinaires récidivantes. Le diagnostic est clinique, confirmé par l´échographie ou l´urétrographie rétrograde, voire l´IRM dans les cas difficiles. La diverticulectomie par voie vaginale est la technique de choix, offrant d´excellents résultats à long terme.

### Etat des connaissances sur le sujet

- *Le diverticule sous-urétral est une affection rare chez les femmes d´âge moyen, pouvant entraîner divers symptômes urogénitaux;*
- *Le diagnostic repose principalement sur l´examen clinique, complété par des examens d´imagerie tels que l´échographie ou l´urétrocystographie rétrograde;*
- *La diverticulectomie par voie vaginale est la méthode de traitement privilégiée, offrant de bons résultats à long terme*.

### Contribution de notre étude à la connaissance

- *Une meilleure compréhension des caractéristiques cliniques et épidémiologiques du diverticule sous-urétral chez les femmes, permettant d´affiner le diagnostic précoce et d´identifier les facteurs de risque spécifiques;*
- *Une confirmation de l´importance du diagnostic clinique, mettant en évidence l´importance de l´examen physique et des antécédents médicaux pour orienter les investigations et les décisions thérapeutiques;*
- *Des données sur les résultats à long terme de la diverticulectomie transvaginale, soutenant son efficacité comme traitement de référence et fournissant des informations sur les taux de réussite, les complications postopératoires et la qualité de vie des patientes*.
